# The impact of UK social distancing guidance on the ability to access support and the health and wellbeing of disabled people during the COVID-19 pandemic: a qualitative exploration

**DOI:** 10.1186/s12889-024-19285-0

**Published:** 2024-06-30

**Authors:** Lucy Nicholls, Alison McKinlay, Rachael Berger, Daisy Fancourt, Alexandra Burton

**Affiliations:** 1 Social Biobehavioural Research Group, Research Department of Behavioural Science and Health, Institute of Epidemiology & Health Care, University College London, 1-19 Torrington Place, London, WC1E 7HB UK; 2 NIHR Policy Research Unit (PRU) in Behavioural Science, UCL Centre for Behaviour Change (CBC), University College London, 1-19 Torrington Place, London, WC1E 7HB UK; 3 Division of Psychology and Language Sciences, Faculty of Brain Sciences, University College London, London, WC1H 0AP UK

**Keywords:** Public health, COVID-19, Physical disability, Wellbeing, Qualitative, Health inequalities

## Abstract

**Background:**

The sudden introduction of social distancing measures in response to the COVID-19 pandemic resulted in significant lifestyle changes for the UK population. People living with physical disabilities were deemed to be at greater risk of complications following COVID-19 infection and were subjected to stricter social distancing guidelines. But gaps remain in our understanding of how the COVID-19 pandemic and associated restrictions affected the ability to access support, health and wellbeing of people with physical disabilities. Such understanding is vital to ensure equitable future pandemic preparedness for people living with physical disabilities.

**Methods:**

We conducted qualitative semi-structured interviews with 31 people living in the UK between May 2020 and January 2022. All participants self-identified as having a physical disability that affected their mobility, sight, or hearing. We analysed the data using reflexive thematic analysis.

**Results:**

Six themes were identified that described the impact of the pandemic on ability to access support, health and wellbeing: (i) adaptations to healthcare provision led to difficulties in managing health and wellbeing; (ii) exacerbations of inequalities in access to public space due to social distancing guidelines; (iii) experiences of hostility from able-bodied people; (iv) loss of social lives and encounters; (v) difficulties maintaining distance from others and subsequent fear of infection and (vi) strategies to support wellbeing and coping when confined to the home.

**Conclusion:**

The COVID-19 pandemic exacerbated existing health and social inequalities experienced by disabled people. The disproportionate impact of the pandemic on service provision and social connections resulted in challenging circumstances for disabled people who faced unmet medical needs, deteriorating health, and at times, hostile public spaces. Disabled people’s experiences need to be incorporated into future pandemic or health-related emergency planning to ensure equality of access to services and public spaces to ensure their health and wellbeing is supported and maintained.

**Supplementary Information:**

The online version contains supplementary material available at 10.1186/s12889-024-19285-0.

## Background

The coronavirus disease (COVID-19) outbreak was declared a pandemic in March 2020, and shortly thereafter, social distancing restrictions were implemented in the United Kingdom (UK) to curb the spread of the virus. This included two-metre distancing rules, limitations on the number of people gathering indoors, closure of non-essential shops and businesses, and mask wearing inside indoor public spaces. At the beginning of the pandemic, many disabled people were deemed to be more at risk of severe complications such as pulmonary or respiratory complications or mortality from COVID-19 infection [1]. In response, the UK government created a classification system to identify certain individuals as ‘clinically extremely vulnerable’, advising them to shield themselves by staying at home and avoiding all face-to-face contact for at least 12-weeks [2]. As the shielding period came to an end however, many disabled individuals struggled to know how best to protect themselves due to insufficient guidance [3].

The social model of disability defines disability as the disadvantage, restriction or exclusion from social participation caused by structural, physical and attitudinal barriers in society [4]. Pre-pandemic research highlights the societal inequalities experienced by people with disabilities, such as exclusion from educational and work opportunities, and being more likely to live in poverty or be diagnosed with a mental health condition [5]. Many of these inequalities were enhanced by the COVID-19 pandemic, with significantly higher levels of loneliness [6, 7], uncertainty, or fear about the pandemic [6, 8], symptoms of anxiety or depression [7, 9], or new or increased frequency of substance misuse and suicidal ideation [9] reported among people with physical disabilities. COVID-19 restrictions also restricted people from being able to maintain a healthy lifestyle through physical activity, which for disabled people resulted in increased pain and decreased physical functioning [10]. Alongside the psychological and physical impacts of social distancing restrictions for disabled people, many experienced disruptions to their usual medical care [11] with nearly half of those experiencing a reduction or cancellation of their medical treatment reporting a worsening of their health during this time [5].

Those with sight or hearing impairments encountered additional challenges navigating public spaces while attempting to follow social distancing regulations. Bubbico et al., 2021 [12] found that facemasks were the greatest challenge to communication among people with hearing difficulties as they hindered the ability to lip read or interpret facial expressions during conversations. Some preventative measures were especially challenging for people with visual impairments, who had difficulty carrying out visually demanding tasks such as handwashing. As such, incidents of handwashing in public spaces were less frequent within this group resulting in an increased risk of infection [13].

Qualitative work conducted with people with disabilities living in the USA during the pandemic highlighted difficulties accessing resources, healthcare, and public amenities. [14, 15], increased anxiety and depression [3], and the need for improved accessibility within vaccination sites and more inclusive COVID-19 communication strategies [1]. A study in New Zealand found that the pandemic exacerbated hegemonic ableist communication, such as health providers adherence to wearing face masks making access to healthcare inordinately difficult for deaf people [16]. Deaf people also experienced disproportionate delays to treatment, unmet medical needs, and prolonged pain [16].

While preliminary investigations have brought to light societal disparities of experiences in different contexts, there is a lack of in-depth research into the effects of the pandemic on the disabled population specifically in the UK. One qualitative study conducted during the first 6-months of the pandemic with people experiencing a wide range of mental, sensory and physical impairments found that disabled people and their families felt abandoned, due to the exacerbation of structural inequalities and unmet needs [17]. There is, however, a lack of in-depth investigation into whether these experiences of inequality continued as the pandemic progressed and as the UK population began to live under different guidelines and levels of restrictions. Capturing these experiences is important to help inform future UK public health strategies and policies so that they sufficiently consider the needs of disabled individuals in future pandemics and, more importantly, in a post-pandemic society. We therefore set out to investigate the impact of the COVID-19 pandemic on the health, wellbeing and social lives of people living with physical disabilities in the UK.

## Methods

This study was part of a larger mixed methods study, which launched in March 2020, investigating the psychological and social impact of the COVID-19 pandemic on different groups of people living in the UK [18], including those with long-term health conditions and mental health problems [19, 20]. As the pandemic and study progressed over time, we expanded the project to include specific groups of people whose voices were missing from the COVID-19 research literature at that time, which included people with physical disabilities. Interviews were conducted between May 2020 and January 2022. The study was approved by the UCL research ethics committee (project ID: 14895/005).

### Recruitment and procedure

Participants were eligible to take part if they were aged 18 or over, could provide informed consent to participate and if they self-reported a physical disability that impacted their mobility, motor skills, sight, or hearing. The study was advertised using a recruitment poster sent via third sector organisations providing support and advice for people with disabilities, social media and a COVID-19 social study email newsletter. The advertisement asked if people had a sensory impairment or physical disability that impacted their mobility or motor skills and if they would be interested in speaking to the research team about their experiences of the pandemic, social distancing and social isolation, including any impacts on mental health, well-being, daily life and the support they had received. Participants were asked to contact a researcher by email or telephone if they were interested and potentially eligible to take part. The researcher then checked eligibility and sent potential participants an information sheet and consent form. Once the consent form was completed, the interview was arranged, and demographic information collected.

**Fig. 1:**
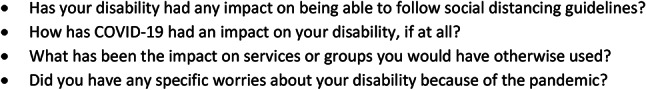
Example questions taken from the interview topic guide

Semi-structured interviews were conducted remotely via telephone or video call by AB, a female senior research fellow with training in qualitative research methods and experience of interviewing people with mental and physical health problems. One interview was conducted by TM (see acknowledgements). The researchers followed a topic guide containing questions and prompts on how the pandemic and associated restrictions had impacted health and wellbeing (See Fig. 1 for example questions and supplementary file 1 for the topic guide). Interviews were audio-recorded with participant permission. At the end of the interview, participants were sent a £10 electronic gift voucher to thank them for their time. Audio-files were then sent for transcription to a UCL approved company via a secure weblink. All data were stored on a secure server (the UCL data safe haven).

### Data analysis

Transcripts were checked for accuracy and deidentified to maintain participant confidentiality before being imported into NVIVO12 for data analysis. We used reflexive thematic analysis to analyse the data [21, 22] informed by a critical realist ontology whereby we aimed to understand and represent the diversity of experience of living with a disability during the pandemic through exploration of personal accounts and stories [23]. Three authors (LN, AM, AB) each coded a separate group of interview transcripts, with all authors following a similar strategy for analysis. AM is a female senior qualitative research fellow with training in conducting research with people experiencing mental health problems. LN is a female research assistant who has previously explored the effects of arts and culture on health, and disability and mental health. The authors read transcripts and coded passages of text pertinent to the research aims. Code labels were selected based on the words that participants used to describe their experiences (for example, ‘absence of care’ and ‘difficulties managing health and wellbeing’), as well as broader categories (i.e., impact of social distancing on service provision). All authors met to discuss code labels that were later grouped and refined based on the combination of codes from each researcher’s dataset. LN then grouped the codes into themes and subthemes. LN, AB, and AM met frequently to discuss their interpretations of the codes and their groupings, to explain and resolve any differences in interpretation and to agree the final themes. Finally, relevant participant quotations were chosen to illustrate each theme and sub-theme. Some quotes have been edited for length and clarity, for example repeated or similar words or phrases within a quote have been removed.

## Results

31 people took part in the study. Participants were aged between 26 and 77 years old, were predominantly White British (26/31) and experiencing mobility problems (25/31). 16/31 participants identified as male. Most participants either lived alone (13/31) or with a partner/ spouse (12/31) and 12/31 participants were unable to work due to their disability. For full participant demographics see Table 1.

**Table 1:** Participant characteristics

Characteristic	Mean (range)/*N*
Age	55 (26–77)
Gender	
Male Female Transgender male	16 14 1
Ethnicity	
White British White Other White Irish Indian Other mixed background	26 2 1 1 1
Living situation	
Alone With partner/spouse With parents With children With housemates	13 12 2 2 2
Marital status	
Married/civil partnership/live with partner Single Divorced/separated Widowed	12 10 8 1
Employment status	
Unable to work due to disability Retired Full time Part time Self-employed Unemployed and seeking work Full time student Missing data	12 5 4 4 3 1 1 1
Qualifications	
Postgraduate Undergraduate Post-16 vocational course A level or equivalent GCSE/CSE/O -levels or equivalent	12 11 4 3 1
Disability*	
Mobility problems Sight impairment Hearing impairment	25 7 6
Mental health condition	
Yes**	13
No	18

*Five participants indicated multiple disabilities (Mobility problems & sight impairment = 2, sight & hearing impairment = 1, mobility & hearing impairment = 1, mobility problems, sight & hearing impairment = 1)

**Reported mental health conditions included depression, anxiety, post-traumatic stress disorder, obsessive compulsive disorder and eating disorder

### Themes

Six key themes were identified during the analysis that represented participant experiences of living with a disability during the pandemic, and the impact of social distancing restrictions on their health, wellbeing and ability to access support: (i) adaptations to healthcare provision led to difficulties in managing health and wellbeing; (ii) exacerbations of inequalities in access to public space due to social distancing guidelines; (iii) experiences of hostility from able bodied people; (iv) loss of social lives and encounters; (v) difficulties maintaining distance from others and subsequent fear of infection and (vi) strategies to support wellbeing and coping when confined to the home. Themes and associated subthemes are presented in Table 2 and described below along with supporting participant quotes.

**Table 2:** Themes and sub themes

Themes	Subthemes
1. Adaptations to healthcare provision led to difficulties in managing health and wellbeing	1.1 Cancelled and delayed appointments and treatments 1.2 Difficulties communicating with health services remotely
2. Exacerbations of inequalities in access to public space due to social distancing guidelines	2.1 Reduced provision of accessibility support 2.2 Queuing for goods and services 2.2 Difficulties navigating public transport and parking
3. Experiences of hostility from able bodied people	
4. Loss of social lives and encounters	
5. Difficulties maintaining distance from others and subsequent fear of infection	
6. Strategies to support wellbeing and coping when confined to the home	6.1 Use of arts and home-based hobbies 6.2 Unhealthy lifestyle behaviors 6.3 Mitigation of social isolation via technology

### Adaptations to healthcare provision led to difficulties in managing health and wellbeing

Many participants experienced a reduced quality of care due to changes in the structure and delivery of health services caused by the social distancing rules. This included appointment and service cancellations, treatment delays and difficulties communicating over the telephone rather than in-person. Many participants described how these changes impacted their ability to manage symptoms, and had a negative impact on their physical health and wellbeing.

#### Cancelled and delayed appointments and treatments

Many participants reported feeling let down by healthcare services and the lack of care received. Participants expressed feeling uncertain in the wake of cancelled or postponed appointments, and that this disruption to care made daily life more challenging:

*“And then appointments keep being cancelled at the last moment, and things being changed, and it’s really disjointed at the moment, and it’s quite hard to get any sense of continuity at all. Which, of course I understand, everyone’s trying to catch up from COVID and stuff, but it’s not making my life very easy to manage.” (female1, age 40–49)*.

An overall increase in demand on the National Health Service (NHS) as a direct result of the pandemic led to longer wait times for referrals which, in turn, delayed disability support:

*“(Social services) referred me to the team that assesses you for continuous NHS health care. Now, normally they should have seen me quite quickly. They’ve still not seen me, still not assessed me. Even though both the social worker and my GP have written to them saying it’s urgent…so it’s quite important that they do assess me. But yes, I’m really upset that they haven’t.” (female2, age 60–69)*.

These delays to treatments throughout the pandemic not only delayed support but resulted in deterioration in their health conditions:

*“I have B12 injections because I can’t absorb it properly… But because of the pandemic, they can’t book me appointments far enough in advance so that I can book the next injection when I have the injection done, which is really complicated and has led me to miss multiple of them…I missed one three months ago. It wasn’t until the next one was due that anybody noticed…My hands were, and still are, worse than they were before I missed one.” (transgender male1, age 20–29)*.

Many participants expressed difficulty in managing pain throughout the pandemic, with some participants attributing this to the closure of leisure and exercise facilities that would have otherwise helped them to maintain good health and wellbeing “*Access to physio was compromised. No gym, no swimming pool, all things I relied on heavily” (female3, age 60–69)*. In addition, a pause in provision of specific pain treatment severely impacted the wellbeing of some participants and caused new symptoms:

*“I used to get nerve root block injections every six months. I’m under pain management team at the Hospital in [Location], and during the pandemic, that was all stopped. So, my pain levels were about three to four out of ten and now they’re six to seven, so that’s really difficult to live with. Now, because my back’s so bad, and my pain levels are so bad, and I’ve got new symptoms, like numbness of my feet…” (female4, age 50–59)*.

#### Difficulties communicating with health services remotely

Participants expressed frustration at being unable to book routine doctors’ appointments during the pandemic due to the implementation of new online booking processes to manage virtual consultations:

*“The local surgery have introduced a new, computerised way of booking appointments, and it’s a real difficulty getting through. You can’t just ring up and say, I’ve got a problem, can I book an appointment with the doctor? The receptionists have been told to tell you to push off and go online. I’ve had to make an appointment about a problem for this week, and it took me about half an hour to get through the computer system to book…” (male1, age 70–79)*.

Many participants described difficulties communicating with healthcare professionals over the telephone as opposed to face-to-face. Conveying symptoms and explaining complex needs to GPs was challenging:

*“They don’t encourage people to go into the surgery. So, it has become a little bit more difficult to see somebody… It’s not good really because I prefer to speak to a doctor face-to-face. It feels like you’re a bit more distant and not having the prompt attention that you should be getting.” (male11, age 70–79)*.

One participant explained that they were no longer able to access in-person appointments which was beginning to “*really eat away at [them]*”, stating that the difficulty of trying to convey symptoms in telephone appointments negatively impacted their wellbeing:

*“…it was the amount of stress that it induced for me, and then I’d wait two or three hours, and if I’m lucky, I get five minutes. I can’t cover anything on the phone, it’s been really hard…I really feel that my overall care has dropped through the floor, which has been quite upsetting.” (female1, age 40–49)*.

A reduction in face-to-face consultations not only affected the wellbeing of those with disabilities but also their physical health. Some participants experienced a deterioration in symptoms or developed health concerns that were left unattended and untreated. One participant with hearing problems described a severe deterioration in their skin condition because they were unable to see a doctor in person:

*“The hearing is the biggest thing, my ears. Because at the beginning of the first lockdown I developed eczema in my ears which affect the hearing aids. And because I couldn’t actually see anybody…and everything was on the phone, which was pretty useless really, I had three really bad ear infections because it just got so bad I couldn’t go to see audiology…so pretty horrendous really.” (female5, age 60–69)*.

### Exacerbations of inequalities in access to public space due to social distancing guidelines

Restricted access to public spaces was experienced by many participants, with some observing inequalities in access once facilities began to open to the general public, difficulties accessing shops and navigating travel. A minority of participants did however observe improvements in accessibility, or experienced positive adaptations to services and spaces.

#### Reduced provision of accessibility support

As guidelines instructed people to work from home or stop work completely, support services to aid those with disabilities to access public spaces became less available, even when facilities were open to the general public:

*“We went to the visitor centre, and all the shops were open, everything was open in [Location], and I asked where the disability assistants were, and they said they were still furloughed. So, that means that there was no access for somebody like me, and that’s the difference. I’ve found that access is worse since the pandemic.” (female4, age 50–59)*.

Further inequalities were experienced in accessing purpose-built facilities that directly supported physical health. One participant described the prolonged closure of inclusive swimming pools despite privately owned leisure centres reopening to the public:

*“I would like to go back swimming, and that has been a bit more difficult because of the pandemic, because many centres…haven’t reopened yet. Because of my disability the swimming pool needs to be warmer, it needs to have a hoist and a lot of these places, which are schools or council places, they have not reopened yet.” (female6, age 50–59)*.

One participant described the adverse effects of the pandemic on previous societal progress that had been made around inclusivity in public spaces:

*“That’s the biggest impact of COVID-19 really, is that all the progress that was made in accessibility seems to have gone back several years…It’s too difficult to go out now. I always find there’s just problems.” (female5 60–69)*.

Some participants did however remark that accessibility had improved for them since the pandemic due to fewer people, shop closures and the subsequent removal of trip hazards:

*“From just going out and about, it was easier for me, because less people out and about. More importantly, because hospitality services were shut, I had less of an issue of tripping over street furniture, board signs and whatnot. So, that was actually a positive for me.” (male3, age 50–59)*.

#### Queuing for goods and services

Queuing outside shops or healthcare services posed a considerable challenge for those with disabilities. exacerbating chronic pain symptoms and causing discomfort. One participant identified queuing as “*one of the biggest problems*” *(female5, age 60–69)* in gaining access to their medications. Many participants stated that no chairs were offered, nor allowances made to accommodate for their disability:

*“They only let you in five minutes before your (hospital) appointment, so we were outside on the street queuing up, and through the cold months, that’s not very pleasant. As I suffer with rheumatoid arthritis and the cold affects the arthritis, then that also wasn’t very good, so I’m having to stand there because there’s no seats.” (Male2, age 50–59)*.

Participants also expressed frustration that adaptations were not put in place for them to simply bypass queues for goods and services:

*“There should have been an awareness, where people with reduced mobility and people in pain, and people with hidden disabilities, should have been able to go to the front of the queue. And it should have been obvious, a sign up, to say, if you’ve got any disabilities…” (female4, age 50–59)*.

The same participant did however acknowledge that their GP surgery had considered those less able to stand and had provided chairs within queuing systems, which contributed towards a feeling of inclusion within the healthcare practice:

*“I think life, in general, is difficult when you’re disabled. So, even getting the vaccine there is a queue, because you’re given a time slot. My surgery is quite good, because the building that they had set up for us, there was chairs in the rows, the zig zag, so there were two points where you could sit down in the queue.” (female4, age 50–59)*.

#### Difficulties navigating public transport and parking

A lack of adequate public transport throughout the pandemic led many participants to feel cut off from everyday life and unable to carry out tasks outside the home. One participant who was blind described the challenges of regular bus routes being reduced or changed, coupled with not being able to access a guide to help navigate these changes because of the social distancing rules:

*“To get anywhere, I rely on public transport, and with that not running on the routes that I need, and even when it does run, things like with the bus station being closed, that means I have to use unfamiliar environments, which means that I’d need guiding. And being guided as a blind person in a safe way, requires being much less than 2-metres away, because you need to take their arm. There are methods of guiding for runners and things, but they don’t really work when you’ve got uneven ground and steps to deal with.” (male4, age 50–59)*.

Many participants echoed feeling frustrated and excluded due to the implementation of pedestrianised streets during the restrictive periods. This significantly reduced access to disabled parking, making it difficult for people to access local facilities.

*“Some of the places that I used to park, one they’ve made quite a lot more of [the Location] pedestrianised, and they seemed to have taken away the blue badges… it all seems small, but actually, it’s just that gradual thing about, oh, actually, this place isn’t sorted for me to be in it.” (female14, age 50–59)*.

### Experiences of hostility from able bodied people

Many individuals encountered difficult or even hostile circumstances throughout the pandemic because of implicit ableism directed at them from members of their community. Some participants described the lack of understanding exhibited by able-bodied people when going about their day because their disability was not perceived as ‘visible’:

*“I’ve noticed a lot of attitudes change, during the pandemic. I’ve had people say to me, you don’t look disabled…you can’t park there, it’s a double yellow line, even though I’ve got a blue badge. And I’ve had people say to me, you’re too young to have a stick.” (female4, age 50–59)*.

One individual who was blind encountered hostility because they needed close physical guidance from others to navigate shops, which was interpreted as breaking the rules:

*“there’s been a lot of hostility to blind people out and about and not social distancing, because we need guiding… At the start it was very, very difficult, and I got through by the seat of my pants to be honest.” (male4, age 50–59)*.

The same participant explained that friends who were also blind had experienced hostility due to requiring additional support when entering shops:

*“People telling them that they shouldn’t be out and about, and just people being really off with people, and not being able to get services anywhere, and being told that only one of them was allowed in a shop at a time, when they actually both needed to go in.”* (*male4, age 50–59)*.

Some even moderated their own behaviour and actions in anticipation of confrontation from able-bodied people when adhering to social distancing rules:

*“The two-metre, then metre distance, I respected it completely, and still try to, but last Saturday, when I was in the convenience store, I had some guy literally right behind me. I could feel his breath on my neck, and I didn’t have the confidence to say, excuse me, could you back off? Because I’ve seen people react, and I can’t be dealing with somebody being angry with me so I let it happen.”* (*female7, age 50–59)*.

### Loss of social lives and encounters

Many participants experienced a decline in wellbeing throughout the pandemic as a result of feeling socially isolated. One participant felt this was due to a loss of confidence because they were socialising with others less often:

*“I think it might be agoraphobia. I’m not sure…I don’t know how to describe the feeling I get, but it’s just, no, I don’t want to go out there. It’s more that I don’t want anybody to see me. I want to remain invisible and anonymous now, which is really bizarre. It’s the complete opposite from what I was.” (female7, age 50–59)*.

Others attributed their sense of isolation to a decrease in social activities as a result of the restrictions:

*“I feel more isolated. I don’t know if lonely is the right word, but I do feel alone. I’m sure there are times when I do feel lonely, but I feel like I’m not included in things…I don’t get invited out, but that feels normal, at this stage. And in my head, the world’s a different place, and as a consequence I am more alone and more isolated than I was.” (female3, age 60–69)*.

Some participants with hearing impairments felt excluded from everyday social encounters due to difficulties communicating with people wearing masks:

*“When everybody was wearing masks, that is a problem, because you can’t lip-read through a mask, so that could make me very tense at times and just feel more isolated, I just couldn’t get through. Now that fewer people are wearing masks, life is easier from that point of view. I’m not shy about asking people to speak louder.” (female8, age 70–79)*.

### Difficulties maintaining distance from others and subsequent fear of infection

Some participants described being concerned about other people’s behaviour, and perceived flouting of the 2-metre distancing rules by others was a recurring theme:

*“The only problem I’ve got is other people. Obviously, I shuffle along, and so, because it’s taking me time, people will get very annoyed with you. And they’re breathing down your neck, they’re not social distancing, they’re right behind you or they’re right alongside you.” (male6, age 60–69)*.

The behaviour of others led to anxiety around catching COVID and this fear of infection led to increased home confinement and a decrease in activities which, in turn, caused adverse health effects:

*“It’s set me back at least two or three years, because I’ve lost all the muscle in the right leg. I was getting stronger and meeting people again. And now I’ve gone back to what I was like four years ago, not seeing anybody, and struggling again to go out, because I’m frightened of catching COVID.” (male7, age 50–59)*.

Some participants were also worried about the potential implications of catching COVID on their health which continued to impact their behaviour around others:

*“The impact has been that I have had to be more careful. If I hadn’t had this disability, I would have been more relaxed with the restrictions and with meeting people and so on. But now the consequences of me getting COVID would be much greater than before I had the accident.” (female6, age 50–59)*.

Wheelchair users described experiencing an invasion of their personal space by members of the public before the pandemic, which had continued during the implementation of social distancing measures. This resulted in undue stress when attempting to maintain the 2-metre distancing rule from others:

*“When you’re in a wheelchair, you’re pretty much invisible, and I’ve never really had a sense of personal space. So, it’s been really difficult for me to go out in my wheelchair, with all the social distancing, because people were terrible beforehand, and it’s still bad…People won’t respect, and it’s so difficult for me to move out of the way. I want people to keep their distance, I don’t want people standing right next to me.” (female1, age 40–49)*.

### Strategies to support wellbeing and coping when confined to the home

Despite the challenges in accessing support and the associated negative impacts of social distancing restrictions on health and wellbeing, many participants also described using strategies to help them cope with isolation and to improve their wellbeing. This included engaging in hobbies and connecting with others via technology. Some unhealthy coping strategies were however also documented including increased alcohol use and overeating.

#### Use of arts and home-based hobbies

Many participants turned to healthy coping strategies during the pandemic as a means of mediating anxiety and easing ill health associated with their disability, including the arts:

*“I’m very restricted physically and most of the time I’m in pain, even when I’m in bed. So that can easily get you down. So I have to make a real effort to concentrate on other, or more positive things. I’m writing a booklet at the moment and still trying to finish my art work. So I keep busy mentally.” (female2, age 60–69)*.

Arts activities such as writing were used as a way of practicing mindfulness or regulating emotion when participants felt lost or upset about their experience throughout the pandemic:

*“It’s been tricky for me, anyway, being in a wheelchair…so I feel like I lost myself, and I think, in truth, I probably was losing myself pre-COVID, but that experience, through the pandemic, I think it really made me realise how much of me has gone… But my writing is something I find very cathartic, so when I’m in that space, I can put anything down, but normally, that can really help my mood.” (female1, age 40–49)*.

One participant explained that participating in art and crafts relaxed them, particularly when stress caused a flare up of physical symptoms:

*“I can be doing something in the house, say for instance mending a switch or something like that, and my hand will be like that with the screwdriver, shaking all over the place. And yet, I can pick up these little diamond things and put them in place, without a tremor, and it really does relax me.”* (*male6, age 60–69)*.

#### Unhealthy lifestyle behaviours

As a result of stressful circumstances caused by the pandemic, some participants adopted unhealthy coping strategies to deal with declining health. When asked whether they had developed any symptoms or habits due to anxiety or stress, one participant acknowledged *“stress eating, which then doesn’t help, because of the dietician and all that jazz that I’m under.” (female9, age 30–39)*.

Others described increased alcohol consumption that had adverse effects on their overall health:

*“It’s just happened recently in the last few weeks, a diagnosis of diabetes 2. That is related to me having become overweight which was something that happened during lockdown. It is in my own unscientific, but anecdotal opinion related to the fact that I drank a lot more during lockdown, which obviously causes an increase in weight. Also, alcohol consumption is related to diabetes 2. I was also taking less exercise.” (Male10, age 60–69)*.

For others, a lack of exercise combined with increased consumption of food and alcohol during the pandemic resulted in weight gain:

*“It’s harder for people who use wheelchairs to lose weight, particularly with somebody of my level of injury that don’t have any control of core muscles and the abdomen. So, it’s going to be tough for me to lose the weight that I’ve put on as a result of inactivity and eating and drinking more, so I think that’s an indirect effect.” (male18, age 30–39).*

#### Mitigation of social isolation via technology

Despite the difficulties of physical exclusion from friends and family, some participants mitigated the isolating effects of staying at home through digital channels of communication. Social media was a popular medium participants used to keep connected:

*“What I will say is if it hadn’t been for Facebook, Twitter and Instagram, I think I would have felt a lot more restricted and a lot more lonely. But because I can be watching The Great British Bake Off, and I’ll follow the Twitter hashtag. I would say I’m more connected now because of social media. And if it wasn’t for social media or FaceTime, I don’t know what I would have done.” (female12, age 40–49)*.

Some even found their social lives expanded as a result of group meetings moving online. Video calls made participating in group activities more accessible which one participant favoured over in-person meetings:

*“My local storytelling group, which I was already finding difficulty in going to because they meet in the evenings, once the pandemic started, decided to have their meetings on Zoom. I discovered that way that I’m good at telling stories on Zoom, which not a lot of storytellers are, at least not at that point*.” *(female8, age 70–79)*.

Video calls negated the need for participants to physically travel to meetings. This made group discussions easier as there were no barriers to accessibility:

*“I didn’t really ever engage with Zoom before. I’m on various different boards, like I’m on my local housing association security board, and days gone by, they’ve asked me to traipse over to somewhere, God knows where for a meeting, whereas now, I can just do it on Zoom.” (male5, age 40–49)*.

## Discussion

Previous research suggests that disabled people reported worse mental health during the COVID-19 pandemic [5] including increased feelings of fear and anxiety [8, 24] and prior to the pandemic, people with disabilities were overrepresented in loneliness and social isolation statistics [25]. Our findings help to understand why mental health may have been so negatively impacted during this time through an in-depth exploration of the experiences of people with disabilities. Overall, we found that pandemic conditions and social distancing restrictions exposed and exacerbated pre-pandemic social inequalities and accessibility issues experienced by people with disabilities. In terms of social inequalities, our findings suggest that loneliness and social isolation may have been exacerbated further due to longer periods of time spent at home because of continued difficulties accessing social spaces and fear of infection, as well as ongoing communication difficulties with service providers and the public attributed to wearing face masks. In terms of accessibility issues, our work also highlights novel and troubling findings related to pandemic experiences, including experiences of hostility and animosity from able-bodied people in public spaces and reports of not being able to access pre-pandemic support when navigating spaces outside of the home, even when restrictions were eased. As a result, some of our study participants felt much of the progress made in accessibility and disability awareness had been reversed due to the pandemic.

Quantitative work has shown that disabled people were twice as likely to report that their access to healthcare had been affected by the pandemic than non-disabled people [26]. Research conducted before the pandemic also identified difficulties accessing healthcare services among people with physical disabilities compared to able bodied people, due to lack of transportation, inaccessible buildings, affordability of travel, and long waiting lists [27, 28]. Our findings provide more nuanced details and show that difficulties accessing healthcare were exacerbated even further as a direct result of adaptations to service provision implemented during the pandemic, such as a lack of in-person appointments, delays to treatment and difficulties conveying health needs over the telephone. This resulted in a worsening of health conditions for some participants.

### Implications for policy and practice

Overall, our findings suggest that there is an urgent need to establish better support systems for disabled people within the healthcare sector, social services, and communities both in the recovery from COVID-19 and in preparation for future pandemics. Healthcare policy must prioritise the needs of disabled people to address ongoing treatment delays and improve the provision of healthcare services to prevent further deterioration in health. In the wake of the pandemic and a decade of austerity, disabled people are currently experiencing unprecedented levels of unmet medical needs [17]. If economic cuts to public and healthcare services continue, those with disabilities are likely to be disproportionately impacted, as already minimal support is stripped back further. In conjunction with the current UK cost-of-living crisis affecting whether people have the means to access dwindling support, the risk to quality of life, and wellbeing of disabled people is considerable. Our study has highlighted the lived experiences of those with disabilities and has shone a light on the lack of attention given to a social model of disability [4] in times of crisis. The social model of disability moves away from a focus on individual impairment, to advocating for a collective responsibility across all sectors of society to identify and implement constructive changes so that barriers to active participation in society are removed for people living with disabilities at all levels. Going forward, this model of disability needs to be fostered to ensure responsibility is shared among government, organisations and the public to identify and remove barriers to access and implement greater social awareness and change towards a more inclusive society both during times of crisis but also in the recovery planning period [29]. For example, disabled people must be at the forefront of healthcare service and social provision planning to ensure any adaptations to service provision meets their needs, with an understanding that people are disabled by barriers in society rather than their own impairments. Public awareness campaigns highlighting experiences of hostility during the pandemic and the impact on disabled individuals need to be implemented, and disability equality training should be regularly provided to those working in public facing services. The adverse impacts felt by disabled individuals during the pandemic also highlight the need for more inclusive community planning and government level commitment to equal access to healthcare and public spaces [30]. Policy makers must learn from the experiences of disabled people during the pandemic to avoid making the same mistakes in future health-related emergencies and to undo the damage created by the pandemic in further exacerbating inequalities among this group of people.

### Strengths and limitations

Our study builds on the findings from quantitative surveys conducted during the pandemic by offering a more detailed qualitative inquiry that helps deepen our understanding of the implications of living with a disability throughout the pandemic in the UK. A strength of this study is the inclusion of people experiencing different and often multiple physical disabilities. However, this broad inclusion criteria may have diluted individual accounts concealing nuances between experiences. In addition, most interviews took place during periods when social distancing restrictions were more relaxed, however, participants reflected on their experiences specifically during lockdowns as well as the longer-term impacts of social distancing guidance, for example continuing to follow guidelines that were no longer in place to keep safe from infection and continued difficulties accessing services. Interviews took place virtually or over the phone, thus excluding those without access to or able to use digital methods of communication. Although we had a large sample size for a qualitative study, most participants were highly educated and there was a lack of ethnic diversity with most participants identifying as White British. Some groups were not represented, such as non-binary individuals. Finally, our recruitment strategy focused on advertising the study via third sector organisations, social media and study newsletters therefore limiting the potential pool of participants who could take part.

## Conclusion

Our study has highlighted the continuing impacts of the COVID-19 pandemic on the health, wellbeing, and inequalities in access to services and public spaces for people with physical disabilities, as well as the exacerbation of pre-existing health and social inequalities. Changes to healthcare provision such as delays to vital treatment, cancelled appointments, and a lack of in-person care led to avoidable deterioration in participants’ health and wellbeing. Social distancing guidelines caused social isolation, significant barriers in navigating public spaces and hostile encounters with able-bodied people outside of the home. The COVID-19 pandemic highlighted a major flaw in the UK’s pandemic planning and preparedness with respect to the needs of people living with physical disabilities. Future crisis planning needs to acknowledge the specific needs of this group and advocate for more inclusive and accessible services and communities to avoid recurring adverse impacts to the health and wellbeing of these groups both during and in the aftermath of subsequent public health emergencies.

### Electronic supplementary material

Below is the link to the electronic supplementary material.

Supplementary Material 1

## Data Availability

The datasets generated and/or analysed during the current study are not publicly available because they consist of transcripts from audio-recorded interviews where participants may discuss sensitive topics and describe multiple health conditions and treatments. The data are available from the corresponding author on reasonable request.
