# Supplementary material for: The impact of UK social distancing guidance on the ability to access support and the health and wellbeing of disabled people during the COVID-19 pandemic: a qualitative exploration
Source: BMC Public Health. 2024 Jun 30;24:1749. doi: 10.1186/s12889-024-19285-0 (PMC11218094; doi:10.1186/s12889-024-19285-0)
Supplement: Supplementary file 1 — Supplementary Material 1 [file 12889_2024_19285_MOESM1_ESM.docx]

**Topic guide**

1. **Ask to describe ‘normal life’**

- Employed? Type of job, hours etc,
- Education/study
- Full time parent or carer?
- Use of any community services/support with disability?
- Who you normally live with, does this change?
- If they have a long-term condition (what condition, when diagnosed, if on or whether they have had treatment)
  - What was current treatment plan? How was it being managed? What was usual routine for appointments/follow-up?
- Whether you would usually have done any type(s) of regular exercise (including walking/gardening)

**UNDERSTANDING AND ADHERENCE TO GUIDELINES**

1. At the moment, are you self-isolating (how long for, reasons for this)?
2. What do you understand by the ‘social distancing’ advice that is being given – what does it mean to you?
3. Have you been able to stick to the social distancing advice that has been given to your group? Please tell us about why/ why not?
4. Has your disability had any impact on being able to follow social distancing guidelines?

**DISABILITIES/LONG TERM CONDITION**

1. How has Covid-19 had an impact on your disability if at all?
2. What has been the impact on any normal appointments?
3. What has been the impact on any other services or groups you would have otherwise used?
4. What has been the impact on any treatment?
5. Have you experienced an impact on any symptoms/side effects?
6. Did you have any specific worries about your disability because of the pandemic?
7. Have you been diagnosed with COVID-19 or suspected you might have had COVID-19? Did this effect your experience of/access to services?
8. How have you felt about [any mentioned changes/impact above]?

**SOCIAL LIFE**

1. How would you describe your social life now that social distancing measures have been brought in because of Covid-19? Please tell us about this

**MENTAL HEALTH**

1. How do you feel about the changes that have been brought about by Covid-19?
2. Have they had any impact on your mental health or wellbeing? Please tell us about these
3. Have you been doing/ planning anything to help with this?
4. Why are you doing/ not doing these things?

**PROSPECTION**

1. Has the pandemic meant that you have any worries for the future?
2. How are these different from the worries you had before?
3. Will this change the way you live your life in future?
4. Has this changed any of your priorities for the future?
